# The Redox Modulating Sonlicromanol Active Metabolite KH176m and the Antioxidant MPG Protect Against Short-Duration Cardiac Ischemia-Reperfusion Injury

**DOI:** 10.1007/s10557-021-07189-9

**Published:** 2021-04-29

**Authors:** Yang Xiao, Karen Yim, Hong Zhang, Diane Bakker, Rianne Nederlof, Jan A. M. Smeitink, Herma Renkema, Markus W. Hollmann, Nina C. Weber, Coert J. Zuurbier

**Affiliations:** 1 Laboratory of Experimental Intensive Care and Anesthesiology, Department of Anesthesiology, Amsterdam Cardiovascular Sciences, Amsterdam UMC, University of Amsterdam, Meibergdreef 9, Amsterdam, The Netherlands; 2 Institut für Herz- und Kreislaufphysiologie, Heinrich- Heine- Universität Düsseldorf, Universitätsstraße 1, Düsseldorf, Germany; 3 Khondrion, Philips van Leydenlaan 15, Nijmegen, The Netherlands; 4 Department of Anesthesiology, Amsterdam UMC, Meibergdreef 9, 1105 AZ Amsterdam, The Netherlands

**Keywords:** Reperfusion injury, Antioxidant, Infarct size, Cytochrome c, Ischemia duration

## Abstract

**Purpose:**

Sonlicromanol is a phase IIB clinical stage compound developed for treatment of mitochondrial diseases. Its active component, KH176m, functions as an antioxidant, directly scavenging reactive oxygen species (ROS), and redox activator, boosting the peroxiredoxin-thioredoxin system. Here, we examined KH176m’s potential to protect against acute cardiac ischemia-reperfusion injury (IRI), compare it with the classic antioxidant N-(2-mercaptopropionyl)-glycine (MPG), and determine whether protection depends on duration (severity) of ischemia.

**Methods:**

Isolated C56Bl/6N mouse hearts were Langendorff-perfused and subjected to short (20 min) or long (30 min) ischemia, followed by reperfusion. During perfusion, hearts were treated with saline, 10 μM KH176m, or 1 mM MPG. Cardiac function, cell death (necrosis), and mitochondrial damage (cytochrome c (CytC) release) were evaluated. In additional series, the effect of KH176m treatment on the irreversible oxidative stress marker 4-hydroxy-2-nonenal (4-HNE), formed during ischemia only, was determined at 30-min reperfusion.

**Results:**

During baseline conditions, both drugs reduced cardiac performance, with opposing effects on vascular resistance (increased with KH176m, decreased with MPG). For short ischemia, KH176m robustly reduced all cell death parameters: LDH release (0.2 ± 0.2 vs 0.8 ± 0.5 U/min/GWW), infarct size (15 ± 8 vs 31 ± 20%), and CytC release (168.0 ± 151.9 vs 790.8 ± 453.6 ng/min/GWW). Protection by KH176m was associated with decreased cardiac 4-HNE. MPG only reduced CytC release. Following long ischemia, IRI was doubled, and KH176m and MPG now only reduced LDH release. The reduced protection against long ischemia was associated with the inability to reduce cardiac 4-HNE.

**Conclusion:**

Protection against cardiac IRI by the antioxidant KH176m is critically dependent on duration of ischemia. The data suggest that with longer ischemia, the capacity of KH176m to reduce cardiac oxidative stress is rate-limiting, irreversible ischemic oxidative damage maximally accumulates, and antioxidant protection is strongly diminished.

**Supplementary Information:**

The online version contains supplementary material available at 10.1007/s10557-021-07189-9.

## Introduction

Over the past few decades, it has become clear that myocardial reactive oxygen species (ROS) formation plays a critical role in myocardial ischemia reperfusion injury (IRI) [1–3]. While there is a mild increase in ROS during ischemia, the largest production of ROS occurs during the initial period of reperfusion [4–7]. During ischemia, the respiratory chain, redox active enzymes, and electron carrier pools become maximally reduced. In parallel, cells become progressively compromised due to ATP depletion, disrupted ion homeostasis, calcium overload, and intracellular acidosis [8]. Consequently, upon reperfusion, the ischemic heart is rapidly flooded with oxygenated blood, and the maximally reduced and impaired mitochondrial electron chain components spill electrons onto oxygen to form superoxide [9]. The net ROS production not only arises from a burst of superoxide from mitochondria upon reperfusion but also due to impairments of the redox ROS-detoxifying system [10]. Preventing the damage caused by ROS production and the aberrant redox system is a promising therapeutic strategy against IR injury.

Recently, sonlicromanol (KH176) has been found to be a potential novel treatment option for mitochondrial-related diseases. Research has shown that sonlicromanol counteracts the biological consequences of complex I dysfunctions in Ndufs4-/- mice (a mammalian model mimicking Leigh disease) by among others scavenging the increased ROS, preventing lipid peroxidation, and increasing lifespan [11, 12]. As a reduction-oxidation modulator, KH176 and its active metabolite KH176m has the potential to diminish the clinical burden of MELAS (mitochondrial myopathy, encephalopathy, lactic acidosis, and stroke like episodes) spectrum disorders [13]. KH176m functions as an antioxidant, directly scavenging ROS, and as a redox modulator, interacting with the peroxiredoxin-thioredoxin system [14]. Based on its mode of action, we hypothesize that KH176m will safeguard hearts from IRI due to its specific antioxidant and redox modulation capacity in cellular systems as well as in vivo including its distribution in hearts in mammals [11, 14].

Up to now inconsistent results regarding the efficacy of antioxidant therapy for cardiac IRI have been reported by both preclinical and clinical studies [5, 15, 16]. One potential reason for this might be the possible dependency upon the duration/severity of ischemia. We and others have previously shown that several cardioprotective interventions (folic acid [17], postconditioning [18]) can be critically dependent upon the duration of ischemia. Such information is currently missing for cardioprotection by antioxidant therapy. This is especially important for translational aspects, when one realizes that cardiac ischemic severity fluctuates largely in the clinical setting [19]. Previous work showed that the irreversible oxidative stress marker 4-hydroxy-2-nonenal is critically dependent on ischemia duration. 4-HNE accumulates during ischemia and peaks between 20 and 30 min ischemia, but does not increase further with prolongation of ischemia [20, 21]. These HNE-protein adducts are unaffected by reperfusion and as such are indicators of irreversible ischemic oxidative stress determined by duration of ischemia. Therefore, in this work, we especially examine cardiac 4-HNE, next to other oxidative stress (3-nitrotyrosine) and apoptosis and autophagy markers, as a potential factor explaining dependency of antioxidant efficacy on ischemia duration for KH176m. Finally, to ensure that ROS does play a role in cardiac IRI, and to compare antioxidant properties, we also evaluated the established antioxidant N-(2-mercaptopropionyl)-glycine (MPG) [22–25] in our IRI model.

In summary, in the present work, we hypothesize that KH176m protects against cardiac IRI for both short and long ischemic insults.

## Methods

### Animals

All animal experimental procedures were approved by the Animal Ethics Committee of the Academic Medical Center, Amsterdam, The Netherlands, and conducted in keeping with the Guide for the Use and Care of Laboratory Animals. C57BL6/N adult mice (male, 10–15 weeks old, 26.7±1.9 g) were purchased from Charles River (Lyon, France). For acclimatization, animals were housed for at least 7 days in standard housing conditions and had food (Teklad global 16% protein rodent diet, #2916, Envigo) and water ad libitum.

### Heart Isolation and Perfusion

The preparations for intact mouse heart have been described in our previous studies [26, 27]. Briefly, mice were anesthetized with sodium pentobarbital (95 mg/kg, intraperitoneally) and injected together with anti-coagulant heparin (15 IU). When needed, extra pentobarbital was administered by intramuscular injection to obtain an adequate depth of anesthesia. After loss of withdrawal reflexes to hind limb toe pinch, mice were intratracheally ventilated with 50% O_2_ and 50% N_2_. Then the aorta was cannulated with a 22G blunt needle, and the heart was immediately perfused. Subsequently, excised hearts were connected to a Langendorff setup and perfused under a constant flow with Krebs-Henseleit (KH) buffer [(in mmol/l), 118 NaCl, 4.7 KCl, 1.2 MgSO_4_, 1.2 KH_2_PO4, 25 NaHCO_3_, 0.5 EDTA, 2.50 CaCl_2_, 5.5 D-glucose, 0.5 L-glutamine, 1 lactate, 0.1 pyruvate, 1% (g/L) albumin – 0.4mM palmitic acid sodium salt, 0.05 L-carnitine, and 30 mU/L insulin]. A water thermo-regulator was used to maintain constant temperature (37°C) for the whole Langendorff system, and KH buffer was filtered by 0.45μm filter and gassed with 95%O_2_/5%CO_2_ (pH=7.4). Cardiac function was assessed by a water-filled balloon inserted into the left ventricle and connected to a pressure transducer. All hearts were subjected to 20-min stabilization, during which balloon volume was adjusted to obtain an initial end-diastolic pressure (EDP) of >2 mmHg and coronary flow (< 4 ml/min) was adjusted to attain an initial perfusion pressure of 75–85 mmHg. Hearts were excluded when developed left ventricular pressure (DLVP) (DLVP= systolic pressure- EDP) was below 80 mmHg, and/or heart rate (HR) was below 280 beats per minute (bpm), and/or arrhythmias after 20 min of stabilization. Rate pressure product was calculated from DLVP × HR. Ischemic contracture onset was defined as the time(s) from the start of ischemia when EDP progressed above 3.0 mmHg, which represents the time that glycolysis stops and ΔG_ATP_ falls below the level needed to support ion pumps and cross-bridges detachment [27]. Delay of contracture is most often associated with improved functional recovery following an ischemic insult.

### Langendorff Protocols (Fig. 1)

**Fig. 1:**
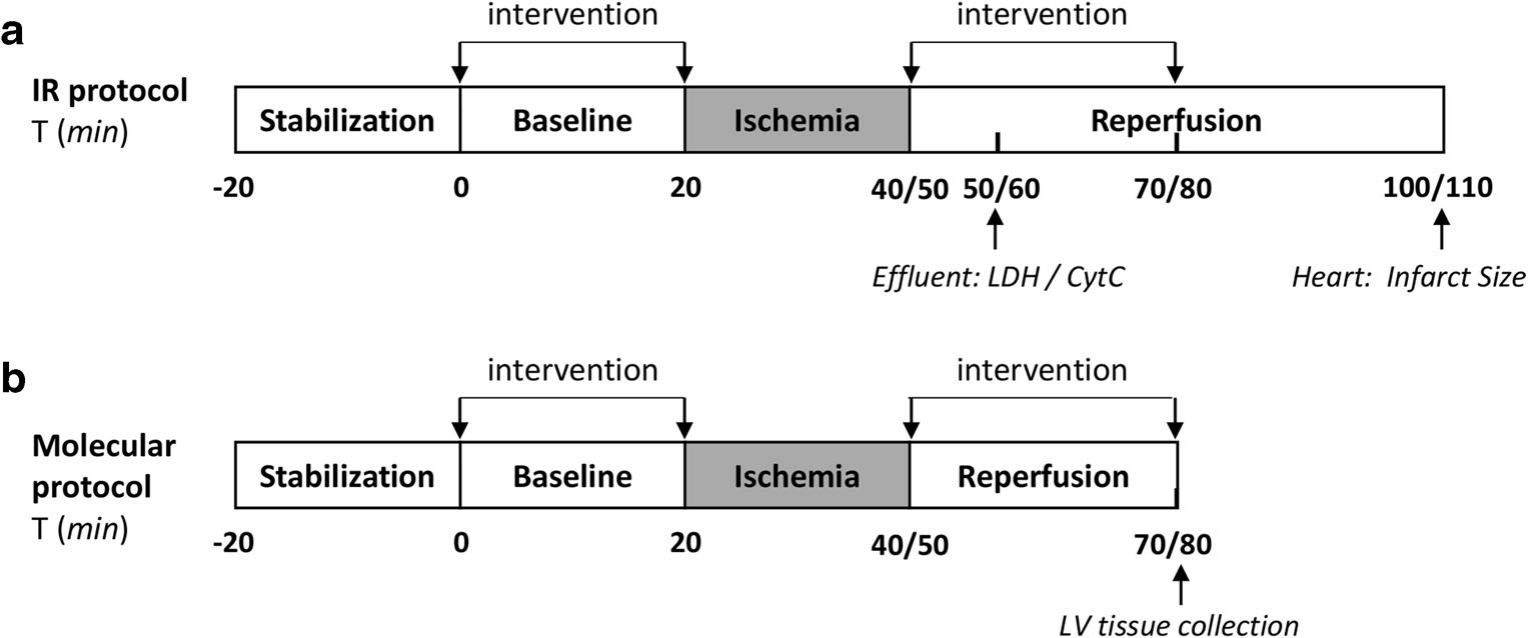
Perfusion protocols for KH176m effects on IR injury. Mouse hearts (*n*=89 for all groups) were stabilized for 20 min, after which 20-min baseline perfusion was started. (**a**) Following baseline, hearts were subjected to either 20-min or 30-min ischemia (I), followed by 60-min reperfusion (R). The coronary effluent was collected at 10-min reperfusion for measuring the LDH and cytochrome c release. Heart was frozen in −20°C immediately at the end experiment for TTC staining. (**b**) Following baseline, hearts were subjected to either 20-min or 30-min I, followed by 30-min reperfusion R, or subjected to 55-min normoxic perfusion as sham group. Left ventricular tissues were homogenized for western blot. Drug delivery started at *t*=0 min baseline and continued for the first 30-min reperfusion; during ischemia drug administration was stopped

1. Pilot experiments: Cellular studies [14] indicated that both 1 μM and 10 μM KH176m reduce oxidative stress. To determine which concentration was optimal for reducing cardiac IRI, we first compared 1μM KH176m, 10μM KH176m, or saline in a limited number of experiments (*n*=4 for control and 1μM KH176m groups; *n*=5 in 10μM KH176m group), according to the protocol provided in Fig. 1.
2. Mild IRI: Following 20-min equilibration, hearts were subjected to 20-min baseline, 20-min global ischemia, and 60-min reperfusion. Hearts were allocated to three groups: control group (saline), KH176m (10μM, dissolved in saline; Khondrion, Nijmegen, The Netherlands) group, and MPG (1mM, dissolved in saline; Sigma-Aldrich, St. Louis, MO, USA) group. Treatments were infused during 20 min prior to ischemia and the first 30 min of reperfusion through a side port above the aortic cannula at 1% of coronary flow (*n*=7 per group).
3. Severe IRI: Similar protocol as for mild IRI, except that now hearts were subjected to 30-min ischemia (*n*=8 for control and MPG groups; *n*=9 in KH176m group).
4. Molecular characterization: Following 20-min or 30-min ischemia and 30-min reperfusion (treated with saline or 10μM KH176m (*n*=8–9 per group)) or following 55-min normoxic perfusion (sham group, *n*=7), left ventricular tissues were immediately homogenized for detection of 4-HNE protein adducts. In addition, we also determined 3-nitrotyrosine (3-NT) as an additional marker of oxidative stress, together with cell death parameters of apoptosis (Bax/Bcl) and autophagy (LC3II/LC3I and p62).

All animals were randomized to morning/afternoon to avoid the influence of circadian rhythm on IR tolerance [28].

### TTC Staining

After 60-min reperfusion, hearts were weighed and stored at −20°C (Fig. 1). 2,3,5-triphenyltetrazolium chloride (TTC, Sigma-Aldrich, St. Louis, MO, USA) staining was performed within 1 week to determine the infarct size. Frozen hearts were cut into 1-mm-thick slices across the short, transverse axis and quickly immersed in 1% TTC solution (pH=7.4) on a shaker (37°C, 300 rpm) for 20 min. Then slides were transferred in order into 4% formalin (pH=7.4) and gently sharked for 2 h at the room temperature. After scanning, infarct size (%IS, percentage of infarct size relative to area of whole heart) was quantified and analyzed with SigmaScan Pro5 software by an investigator not aware of treatment allocation.

### LDH Activity Measurement

Lactate dehydrogenase (LDH) activity is an important marker for cell death. Coronary effluent was collected at 10 min of reperfusion (Fig. 1) and immediately stored at −80°C until further processing. LDH activity was measured by spectrophotometry at 340 nm and 25°C blindly. Activity was determined by the rate of NADH oxidation in assay buffer (containing pyruvate as substrate). LDH activity in coronary effluent was corrected by heart wet weight and coronary flow.

### Cytochrome c ELISA Analysis

Rat/mouse cytochrome c immunoassay (MCTC0, R&D systems) was used to determine cytochrome c concentration in coronary effluent collected at 10min of reperfusion (Fig. 1) according to the manufacturer’s instructions. In brief, after adding conjugate to each well, standards or samples were added and incubated for 2 h at room temperature on the shaker (300 rpm). Each well was aspirated/washed five times, substrates solution added, and incubated for no more than 30 min at room temperature in the dark. After stopping reaction, optical density was read at 450nm and corrected to 540nm. Cytochrome c concentration was corrected by heart wet weight and coronary flow.

### Tissue Homogenization and Western Blotting

After molecular characterization protocol, left ventricular tissue was quickly minced in ice cold homogenization buffer containing 0.02 M HEPES, 0.25 M sucrose, 1X HaltTM protease and phosphatase inhibitor (Thermo Fisher, #78442), and 2% mercaptoethanol to prevent protein oxidation. Tissues were homogenized in potter S homogenizer at 1200 rpm/min, supplemented with 0.5% Triton X-100 on ice for 10min following which samples were sonicated for 5 s and centrifuged for 2 min (10,000g, 4°C). Supernatant samples were aliquoted and stored in −80 °C for further processing.

Lowry assay was used to determine protein concentration. Western blotting was conducted as described previously [29]. In Brief, 18 μg protein per sample was electrophoresed on a 4–12% precast polyacrylamide gel (Bio-Rad, #345-0125) and transferred to polyvinylidene fluoride (PVDF) membrane. After incubation with blocking buffer (Odyssey, #927-70001) for 1 h, membrane was probed with primary antibody for 4-HNE (1:1000; Abcam ab46545), 3-nitrotyrosin (1:500; Abcam ab61392), Bcl2 (1:1000; CST #2876), Bax (1:200; Santa Cruz P-19, sc-527), LC3 (1:500; NB100-2220), and P62 (1:1000; Abcam ab91526) over night at 4°C. Membranes were washed with PBS-Tween20 and incubated with the complementary secondary fluorescence antibody (IRDye, Li-COR, Lincoln, USA, 1:5000; goat anti-rabbit #926-68071/926-32211 or goat anti-mouse #926-32210) for 1h at room temperature. Membranes were washed again and scanned with Odyssey scanner (LI-COR). Membrane were subjected to Coomassie blue (Bio-Rad # 1610786) staining to confirm equal loading of protein.

### Statistical Analysis

All results are expressed as mean ± SD. *N* is the number of animals used unless stated otherwise. For the IR experiments, an initial *n*=7 was determined to be able to detect a clinically relevant increase of 30% in RPP recovery with an *α*=0.05, SD=8, and a power of 0.8. Shapiro-Wilk was used to test the normality distribution of data. One-way ANOVA was performed when data was normally distributed, and multiple comparison was followed by an LSD or Dunnett T3 post hoc test. Non-normally distributed data was analyzed with independent sample Kruskal-Wallis test, unless otherwise stated. Statistics were conducted using IBM SPSS statistics version 26 (International Business Machines Corp., Armonk, NY, USA). Figures were made in GraphPad Prism 8.0 (GraphPad Software, Inc., La Jolla, CA, USA). In all tests, significance was accepted for *P* < 0.05.

## Results

### Ten micromolar KH176m Showed Potential for Cardioprotection

**Fig. 2:**
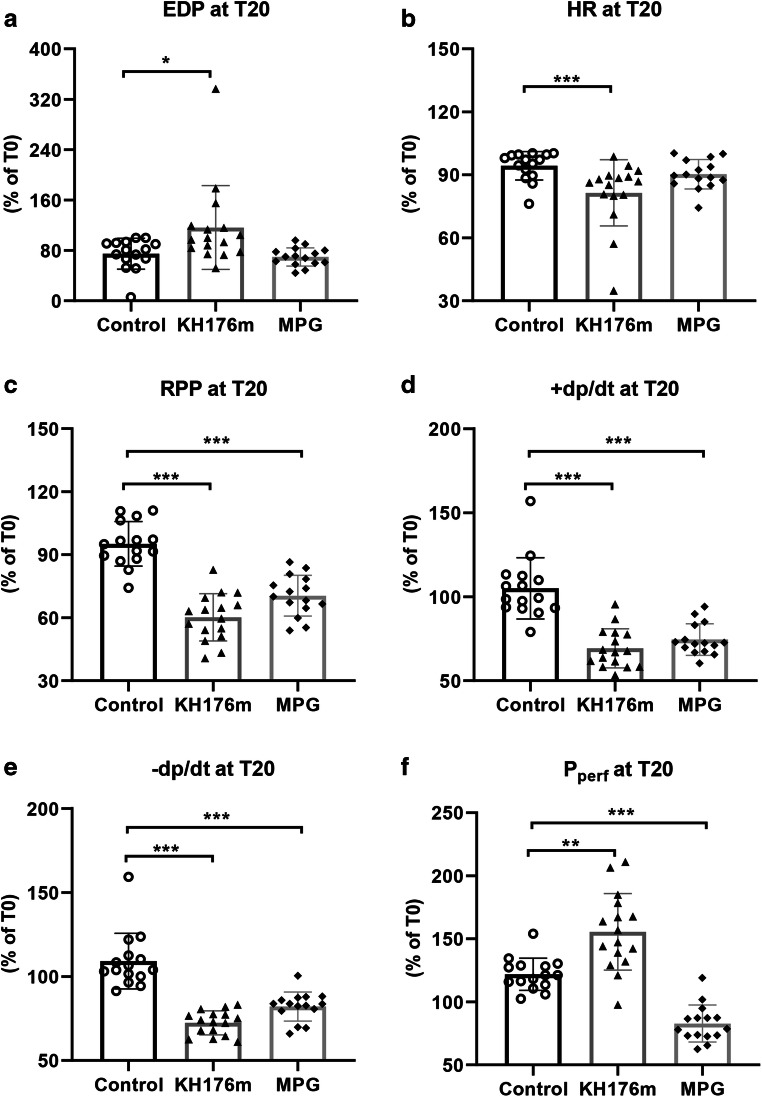
Cardiac mechanical parameters following 20-min perfusion with cardioprotective agents. Heart was subjected to saline (as control group), KH176m (10 μM), or MPG (1mM) for 20min prior to ischemia. For all parameters, the change at *T*=20 min relative to the value at *T*= 0 min is depicted. (**a**) End-diastolic pressure (EDP), (**b**) heart rate (HR), (**c**) rate pressure product (RPP), (**d**) maximum contraction rate of the left ventricle (+dp/dt), (**e**) maximum relaxation rate of the left ventricle (-dp/dt), and (**f**) perfusion pressure (Pperf). *P<0.05, **P<0.01, ***P<0.001 vs control group

At 10 μM KH176m significantly inhibited cell death as reflected by reduced LDH release (control 0.95 ± 0.53, 10 μM KH176m 0.17 ± 0.21 U/min/GWW, *P*<0.05, Fig. 2c) and cytochrome c release (control 988.0 ± 380.7%, 10 μM KH176m 192.4 ± 178.9 ng/min/GWW, *P*=0.055, Fig. 2d). As a lower dose (1 μM) failed to rescue IR injury induced by mild ischemia (supplementary Fig. 1), 10 μM KH176m was selected for all following experiments.

### Ten micromolar KH176m and 1mM MPG Suppressed Mechanical Function During Normoxic Perfusion

Cardiac physiological parameters at baseline T0 (time= 0 min) are summarized in supplementary Table 1, showing no differences among groups. Hereafter, hearts were normoxically perfused and subjected to different treatments (saline, 10μM KH176m, or 1mM MPG) for 20 min. Compared to control group, both KH176m and MPG decreased all variables of mechanical function during normoxic perfusion, except for end-diastolic pressure (Fig. 2). Interestingly, KH176m and MPG displayed opposite effects on resistance of coronary vasculature. At constant flow, KH176m developed higher perfusion pressure (155.5 ± 30.4%, *P*<0.01), whereas MPG treatment resulted in decreased perfusion pressure (82.8 ± 14.7%, *P*<0.01), as compared to the control group (121.9 ± 12.8%, Fig. 2f).

### Ten micromolar KH176m and 1mM MPG Both Reduced Mild IR Injury

#### Cardiac Function Variables

**Fig. 3:**
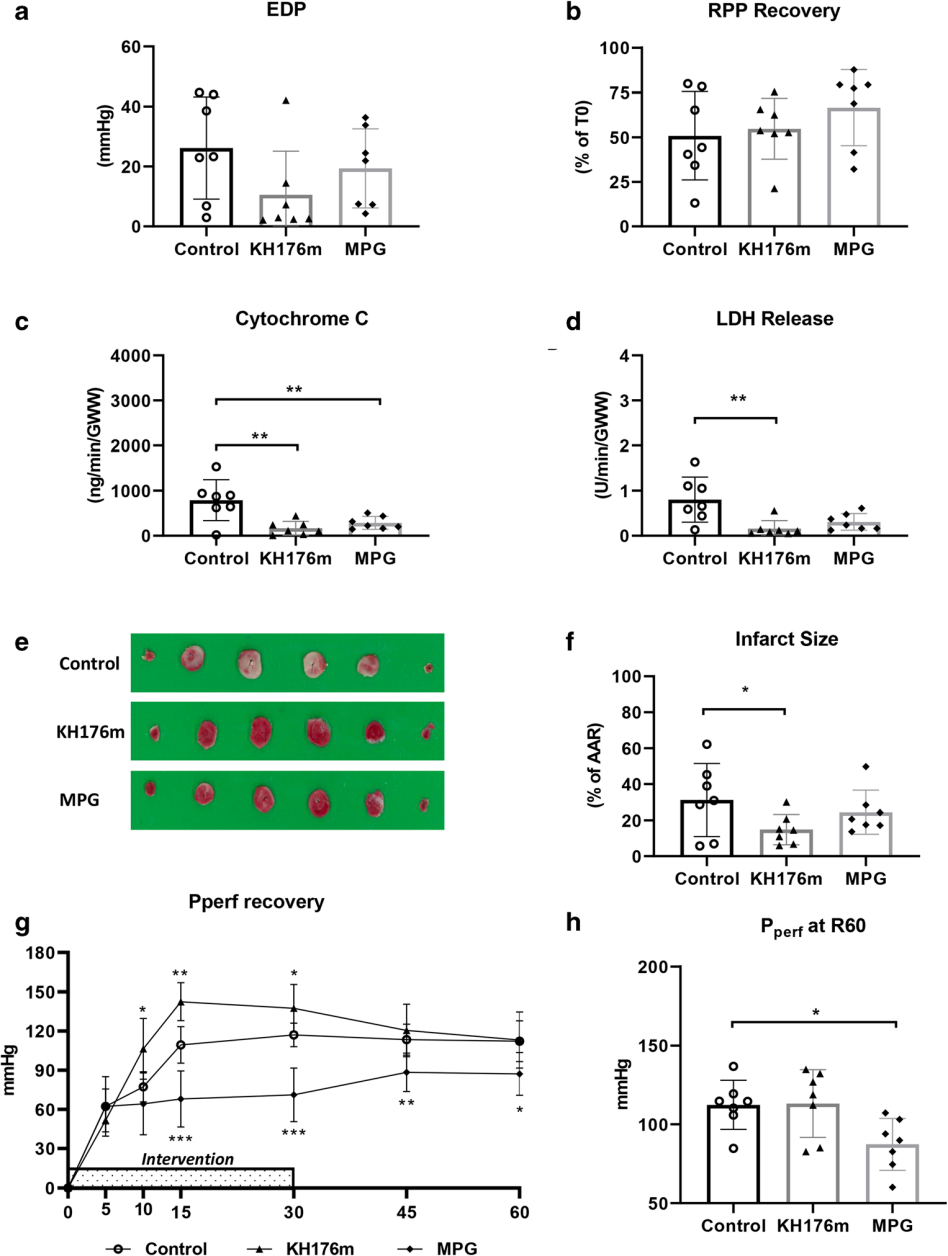
Cardiac function and cell death following a mild ischemic insult. Hearts were subjected to mild (20 min) ischemia followed by 60-min reperfusion, administrated with saline (as control group), KH176m (10 μM), or MPG (1mM). (**a**) EDP at 60-min reperfusion and (**b**) RPP recovery at 60-min reperfusion related to *T*= 0 min. (**c**) Cytochrome c release at 10-min reperfusion, normalized to coronary flow and heart wet weight; (**d**) LDH release at 10-min reperfusion, normalized to coronary flow and heart wet weight; (**e**) image of TTC staining; (**f**) infarct size related to AAR; (**g**) perfusion pressure recovery during 60-min reperfusion; and (**h**) perfusion pressure at 60-min reperfusion. *P<0.05, **P<0.01, ***P<0.001 vs control group

End-diastolic pressure (EDP) at the end of reperfusion amounted to 26.2 ± 17.1 mmHg and was unaffected by treatment (Fig. 3a). RPP recovered to 55 ± 17% in the 10 μM KH176m group and 67 ± 21% in the 1mM MPG group, similar as observed for the control group (51 ± 25%, *P*>0.05, Fig. 3b).

#### Cell Death Variables

Specified areas of risk and infarction as determined by planimetry (SigmaScan Pro5 software) are summarized in supplementary Table 2. Following 20 min of ischemia, KH176m (10 μM) strongly reduced mitochondrial damage (CytC: 168.0 ± 151.9 vs 790.8 ± 453.6 ng/min/GWW, 10 μM KH176m vs control) and cell death (LDH: 0.2 ± 0.2 vs 0.8 ± 0.5 U/min/GWW; %IS: 15 ± 8 vs 31 ± 20%, 10 μM KH176m vs control). MPG (1mM) only reduced mitochondrial damage (CytC: 284.7 ± 141.4 ng/min/GWW), but not LDH release or %IS (Fig. 3c–f).

#### Coronary Vascular Resistance

Upon reperfusion, KH176m (10 μM) significantly increased vascular resistance, as reflected by elevated perfusion pressure. Halting KH176m administration quickly restored vascular resistance to levels similar as control group. MPG (1 mM) caused significant vasodilation during treatment (at 30 min reperfusion perfusion pressure: 71.2 ± 20.6 vs 117.0 ± 9.1 mm Hg, 1 mM MPG vs control), whereby vasodilation persisted after halting MPG administration (at the end of reperfusion: MPG 87.2 ± 16.4 mmHg, control 112.3 ± 15.6 mmHg, *P<0.05*, Fig. 3g and h).

Combined, 10 μM KH176m offered strong protection against a mild ischemic insult of the heart, often superior to the classical antioxidant MPG at a much higher concentration (1 mM). KH176m increased coronary vascular resistance, whereas MPG decreased vascular resistance, both before and after an ischemic insult.

### Prolonged Ischemic Insult Abolished 10 μM KH176m and 1mM MPG Protection

Extending the duration of ischemia from 20 to 30 min doubled IRI for all outcome parameters, indicating increased severity of the ischemic insult.

#### Cardiac Function Variables

**Fig. 4:**
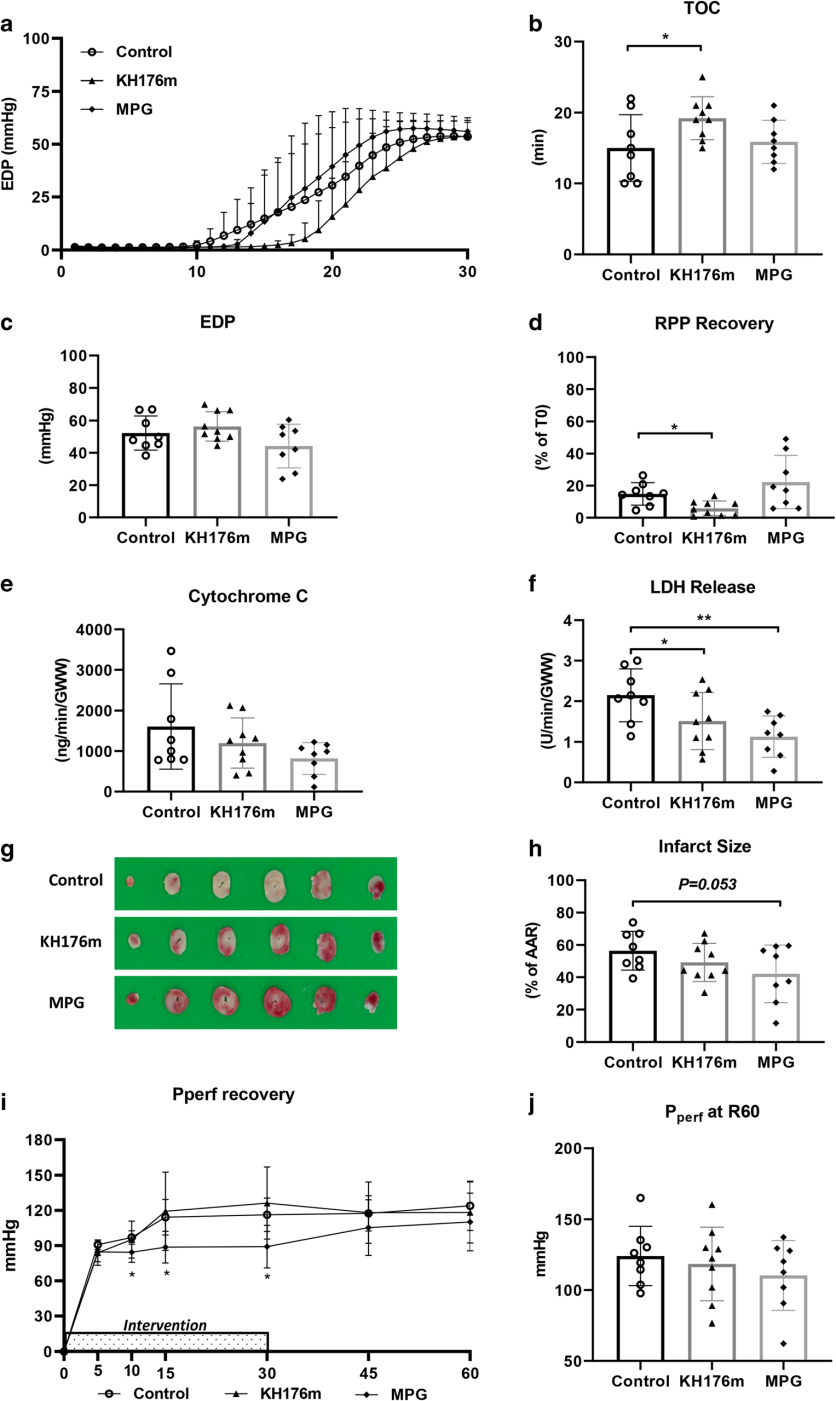
Cardiac function and cell death following a severe ischemic insult. Hearts were subjected to severe (30 min) ischemia followed by 60-min reperfusion, administrated with saline (as control group), KH176m (10 μM), or MPG (1mM). (**a**) Contracture development during ischemia; (**b**) time to contracture (TOC); (**c**) EDP at 60-min reperfusion; (**d**) RPP recovery at 60-min reperfusion related to *T*= 0 min; (**e**) cytochrome c release at 10-min reperfusion, normalized to coronary flow and heart wet weight; (**f**) LDH release at 10-min reperfusion, normalized to coronary flow and heart wet weight; (**g**) image of TTC staining; (**h**) infarct size related to AAR; (**i**) perfusion pressure recovery during 60-min reperfusion; and (**j**) perfusion pressure at 60-min reperfusion. *P<0.05, **P<0.01 vs control group

Contracture development and time-of-contracture (TOC) during ischemia are depicted in Fig. 4a and b, respectively. Ten micromolar KH176m treatment significantly delayed contracture development during ischemia, whereas 1 mM MPG was without effect. However, 10 μM KH176m was unable to improve EDP or %RPP at the end of reperfusion (Fig. 4c and d). One millimolar MPG was also unable to protect the heart against IR-induced cardiac dysfunction.

#### Cell Death Variables

For the long ischemia duration, 10 μM KH176m was now unable to reduce mitochondrial damage (Fig. 4e) and infarct size (Fig. 4g and h); only for LDH release a protective effect was observed (10 μM KH176m 1.5 ± 0.7, control 2.2 ± 0.7 U/min/GWW, *P*<0.05, Fig. 4f). Also, 1 mM MPG only significantly reduced LDH release (1 mM MPG 1.1 ± 0.5, control 2.2 ± 0.7 U/min/GWW, *P*<0.01, Fig. 4f), with a trend to decrease %IS (1 mM MPG 42 ± 18%, control 56 ± 12%, *P*=0.053, Fig. 4g and h).

#### Coronary Vascular Resistance

No effects of 10 μM KH176m on coronary vascular resistance were observed following reperfusion of severe ischemia, not during or after 10 μM KH176m administration. In contrast, 1 mM MPG still induced vasodilation during, but not after, administration (Fig. 4i and j).

Summarized, with severe ischemia, 10 μM KH176m lost most of its protective effect on cardiac IRI and coronary vascular resistance during reperfusion. Similar reduced protective properties were observed for 1 mM MPG, although to a lesser extent.

### Ten micromolar KH176m Reduced Oxidative Stress Following Mild IR Injury

To further clarify whether the cardioprotection of 10 μM KH176m in mild but not severe IR injury is associated with the retain of its antioxidant effects, left ventricular tissues were used for oxidative stress and cell death detection. Cardiac physiological parameters at baseline T0 (time= 0 min) are summarized in supplementary Table 3, showing no differences among 5 groups.

#### Oxidative Stress

**Fig. 5:**
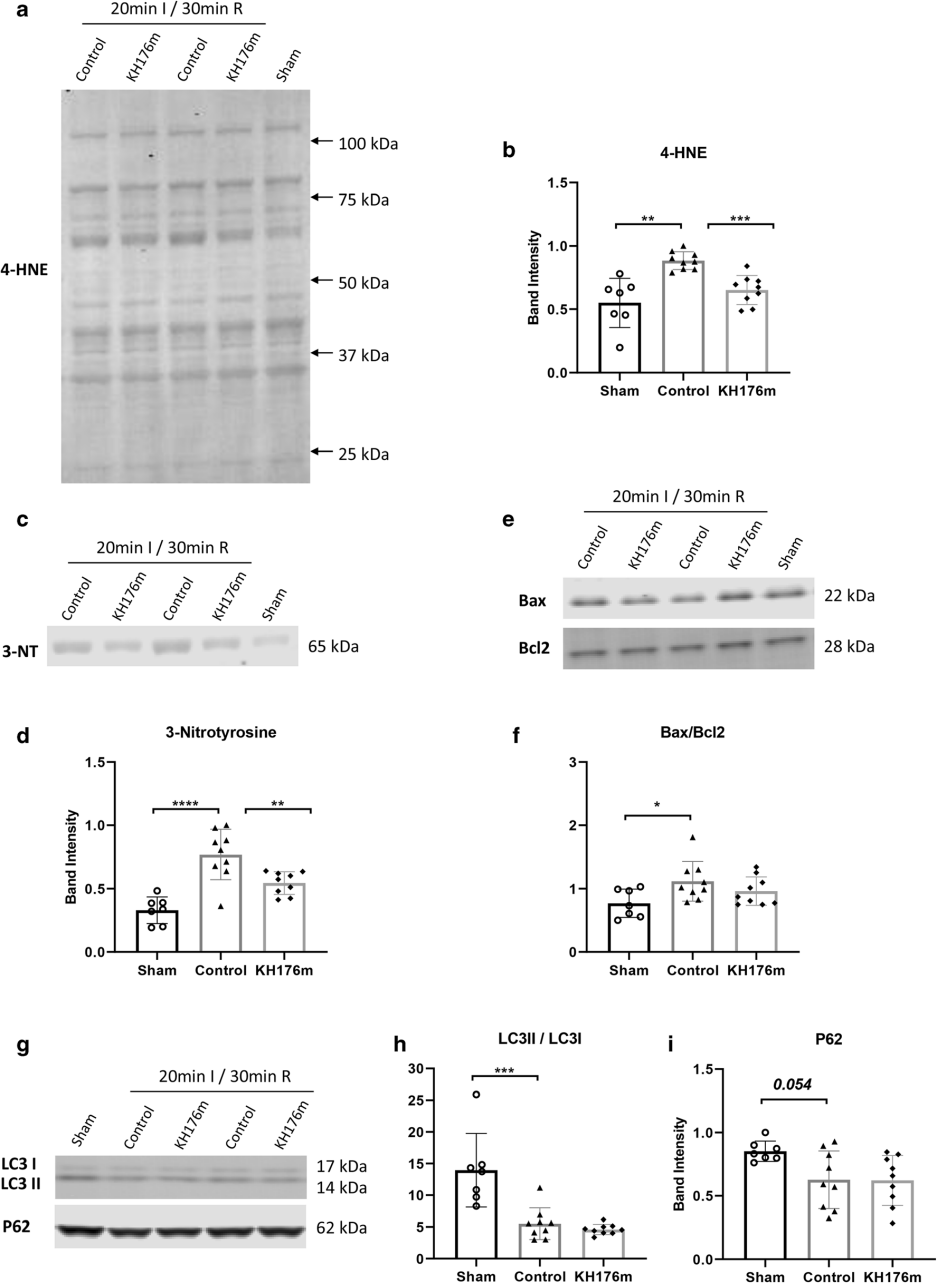
Oxidative stress, apoptosis, and autophagy following a mild ischemic insult. Hearts were subjected to mild (20 min) ischemia followed by 30-min reperfusion, treated with saline (as control group) or 10μM KH176m or subjected to 55-min normoxic perfusion as sham group. (**a**, **b**) Representative immunoblots and analysis of 4-HNE. (**c**, **d**) Representative immunoblots and analysis of 3-NT. (**e**, **f**) Representative immunoblots and analysis of Bax and Bcl2. (**g**, **h**) Representative immunoblots and analysis of LC3II/LC3I and P62. *P<0.05, **P<0.01, ***P<0.001, ****P<0.0001

After 20-min ischemia, oxidative stress significantly increased compared to sham (no IR injury) group. Ten micromolar KH176m strongly decreased lipid peroxidation parameter 4-hydroxynonenal (4-HNE) compared to control group (10 μM KH176m 0.651 ± 0.115, control 0.884 ± 0.071, *P*<0.001, Fig. 5a and b). Ten micromolar KH176m also decreased the 3-nitrotyrosine (a biomarker of nitrogen free radical species modified proteins) level compared to control group (10 μM KH176m 0.545 ± 0.090, control 0.770 ± 0.199, *P*<0.01, Fig. 5c and d).

#### Cell Death

The pyroptosis, apoptosis, and autophagy parameters were examined for understanding the role of different kinds of cell death in KH176m’s protection. Nod-like receptor protein-3 (NLRP3), an important molecule in inflammasome formation and pyroptosis activation, was not detectable in our heart tissues. There was increased apoptosis (increased Bax/Bcl2 ratio, Fig. 5e and f) and increased autophagy degradation (decreased LC3II/LC3I ratio and a trend of decreased P62, Fig. 5g–i) following 20-min IR injury compared to sham group. However, 10 μM KH176m treatment was without significant effects on any of above parameters, indicating that 10 μM KH176m cardioprotective effect cannot be ascribed to changes in apoptosis or autophagy.

Combined, the cardiac protection of 10 μM KH176m against a mild ischemic insult was associated with decreased oxidative stress, without affecting apoptosis or autophagy.

### Prolonged Ischemic Insult Abolished 10 μM KH176m’s Antioxidant Effects

#### Oxidative Stress

**Fig. 6:**
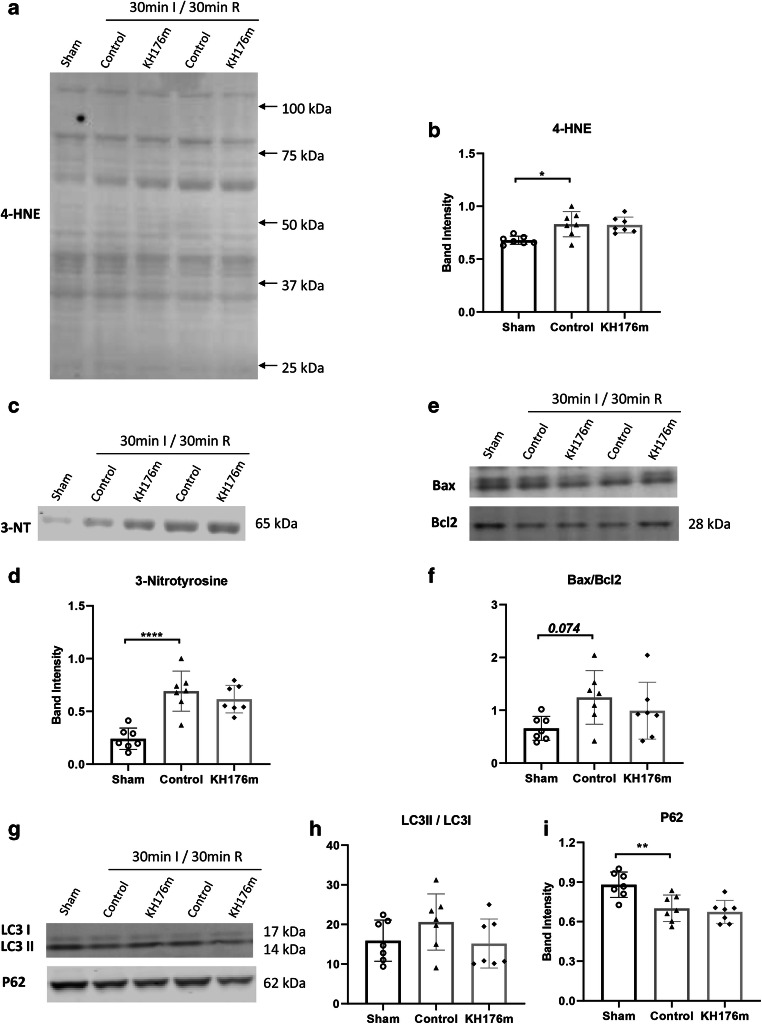
Oxidative stress, apoptosis, and autophagy following a severe ischemic insult. Hearts were subjected to severe (30 min) ischemia followed by 30-min reperfusion, treated with saline (as control group) or 10μM KH176m or subjected to 55-min normoxic perfusion as sham group. (**a**, **b**) Representative immunoblots and analysis of 4-HNE. (**c**, **d**) Representative immunoblots and analysis of 3-NT. (**e**, **f**) Representative immunoblots and analysis of Bax and Bcl2. (**g**, **h**) Representative immunoblots and analysis of LC3II/LC3I and P62. *P<0.05, **P<0.01, ****P<0.0001

After 30min ischemia, similar to 20-min ischemia, oxidative stress significantly increased compared to the sham group. Ten micromolar KH176m was without effects on 4-HNE (10 μM KH176m 0.823 ± 0.075, control 0.831 ± 0.119, *P*>0.05, Fig. 6a and b) or 3-NT (10 μM KH176m 0.616 ± 0.131, control 0.692 ± 0.190, *P*>0.05, Fig. 6c and d) compared to control group.

#### Cell Death

Following 30-min ischemia, Bax/Bcl2 ratio was unaltered, whereas autophagy was increased as indicated by decreased P62 (Fig. 6e–i). However, 10 μM KH176m treatment showed no effects on these apoptosis and autophagy parameters.

Combined, the loss of protection of 10 μM KH176m against the long ischemic insult was associated with insufficient antioxidant activity to reduce cardiac oxidative stress markers, without relevance to apoptosis or autophagy.

## Discussion

We here present several observations on the role of the ROS-redox modulator KH176m and the antioxidant MPG during ischemia reperfusion injury in the isolated mouse heart. By focusing on both mild and severe injury, we observed that (1) the optimal dose (10 μM) KH176m protected against mild IR injury, (2) KH176m reduced mitochondrial damage and cell death when subjected to a short, 20 minute, period of ischemia, which related to its antioxidant efficiency, (3) KH176m and MPG had limited protective effects against cardiac IRI with prolongation of the ischemic insult, (4) KH176m, but not MPG, delayed the development of contracture during ischemia, and (5) 10 μM KH176m increased coronary vascular resistance during baseline perfusion and reperfusion following a short, but not long, ischemic insult. The here reported dependency on ischemia duration for cardioprotective efficacy of antioxidant treatment against cardiac IRI ex vivo illustrates the need for proper titration/dosing of antioxidants in clinical settings where large variations in ischemic insult duration are commonly present.

Khondrion’s clinical stage lead compound KH176 (sonlicromanol) was subjected to phase I [30] and phase IIA [13] clinical trials developed for mitochondrial-related diseases based on its intracellular reduction-oxidation modulation. A phase IIB study is currently recruiting patients. Clinical trial results established that in human blood samples following sonlicromanol dosing, the ratio of the active KH176m metabolite versus active KH176 approximates 0.3. Concentrations of KH176m after 100 mg BID of sonlicromanol reached Cmax values of about 0.5 μM. Whereas both compounds have similar redox regulation, KH176m has improved ROS scavenging properties as compared to KH176 [14]. Our current study shows that KH176m also protects the ex vivo intact heart against mild acute IRI. The KH176m concentration of 10 μM used in this study however is much higher than the maximum blood concentration reached during the chronic 100 mg BID sonlicromanol clinical trials (phase I and IIA). However, when applying allometric scaling, dosages for the mice are commonly 12 times higher than needed for humans, indicating that the 10 μM mouse dose will translate into 0.8 μM human dose necessary to reduce human cardiac I/R injury; more data in humans is needed to adequately address this question.

### KH176m Versus MPG

KH176m has direct ROS scavenging capacity and has shown to activate the thioredoxin/peroxiredoxin enzyme complex. Previous work demonstrated the protective effect of peroxiredoxin against oxidative stress-induced cardiac cell death [31]. N-mercaptopropionylglycine (MPG) is a sulfur-containing exogenous antioxidant with direct ROS scavenging capacity and protective effects on cardiac IRI [32]. Although both KH176m and MPG protected against mild cardiac IRI, our data demonstrated that KH176m had a stronger cardioprotective activity than MPG, especially when one considers that MPG was administered at a 100 x higher dosage than KH176m in the present study. This improved protective effect of KH176m is possibly due to the additional ROS attenuating effect of KH176m as a peroxiredoxin-thioredoxin activator. KH176m delayed the development of ischemic contracture, whereas MPG did not. Since the onset of ischemic contracture was a read-out of a critical low ratio of ATP synthesis over ATP demand, resulting in a critically depressed ΔG_ATP_, KH176m’s special properties (thioredoxin/peroxiredoxin complex activation) might delay the development of a low energy status during ischemia. How exactly the peroxiredoxin-thioredoxin system protects the energy status during ischemia is unknown and will deserve further research. One potential mechanism could be a delay in mitochondrial depolarization, thereby attenuating the reversal of the mitochondrial F_o_F_1_ ATP synthase to an ATPase and thus slowing ATP breakdown during ischemia required to prevent mitochondrial depolarization [33].

The antioxidant MPG was associated with a decrease in coronary vascular resistance, whereas KH176m resulted in the opposite reaction, i.e., increased vascular resistance. Interestingly, these vascular effects were independent of I/R-induced ROS signaling, since they were already present during baseline perfusion of the hearts. This partly illustrates the well-known signaling function of ROS in the non-stressed healthy condition. The decrease in vascular resistance with MPG has been demonstrated before [34] and is likely related to a diminished ROS-NO interaction, facilitating NO-mediated relaxation of smooth muscle constriction and therefore vasodilation [35]. The increase in vascular resistance with KH176m is more difficult to explain. Possibly, the activation of the thioredoxin/peroxiredoxin system by KH176m shuttles more NADPH into this system, away from the nitric oxide synthases, thereby reducing NO production and NO-mediated vasodilation. Further research is needed to elucidate the molecular mechanism of the observed coronary vasoconstriction by high dose of KH176m.

### Antioxidant Therapy in Mild Versus Severe Ischemia

The present work illustrates that antioxidant efficiency against cardiac IRI is critically dependent on severity of the ischemia, i.e., ischemia duration. This dependency on ischemia duration has now been reported for several other cardioprotective interventions, such as folic acid administration [17], postconditioning [18], cyclophilin D ablation and cyclosporine A administration [36], sevoflurane administration [37], and very recently for a humanin analogue [38]. Our present study highlights this dependency now also for antioxidant therapy.

Our data demonstrated that the protection of KH176m against cardiac IRI is associated with decreased oxidative stress markers following short ischemia, but that protection is lost with long ischemia accompanied with the inability to reduce cardiac oxidative stress markers. Previous work in isolated rat heart has shown that the irreversible oxidative stress markers develop during ischemia, reaching maximal levels between 20- and 30-min ischemia without further increases when extending ischemia > 30 min and that reperfusion, or applying antioxidant only during reperfusion, is without effect on these oxidative stress marker [20, 21]. Taking these previous works into account, we suggest that the amount of KH176m present within the heart at the start ischemia is sufficient to prevent peak accumulation of oxidative stress levels during short, but not during long ischemia, such that no reduction in these irreversible stress markers is observed at 30-min reperfusion, the damage is done and protection is lost. Such a scenario is also commensurate with our results showing that the loss of protection seems to be more pronounced for the KH176m treatment, being present in a lesser amount (10 μM) at start ischemia than MPG, that was perfused at 1 mM (see Fig. 4). Thus, our data together with [21] indicate that reduction in oxidative stress developing *during ischemia* by antioxidants is likely a major contributor to its potential to protect against cardiac IRI. Previous literature already indicated that ischemic oxidative stress, next to reperfusion oxidative stress, is a major player in cardiac IRI [39]. It should hereby be realized that we did not examine reversible ROS levels, but irreversible ROS damage as depicted by 4-HNE. When reperfusion ROS had been important for cardiac IRI in our model, the continuous presence of our antioxidants in the perfusate during reperfusion should have also protected against long ischemia. That reperfusion ROS is less likely to be a major causal factor for acute cardiac IRI and is in support of recent findings that reperfusion ROS occurs after, not before, mPTP opening [40]. The oxidative stress during ischemia may exert its effects through increasing the sensitivity of the mPTP for opening at the moments of early reperfusion [41]. Further experiments will be necessary with increasing dosages of KH176m present in the heart before ischemia and/or not applying KH176m during reperfusion to test the hypothesis that it is indeed the oxidative stress generated during ischemia that mainly determines acute cardiac IRI and that loss of antioxidant protection with long ischemia is caused by the loss of effective concentrations of the antioxidant within the ischemic heart to prevent oxidative stress levels to reach irreversible maximum concentrations.

In conclusion, 10 μM of the active compound KH176m of the phase IIB clinical stage active drug sonlicromanol protects the ex vivo intact hearts from mild IR damage, probably through its ability to attenuate the generation of oxidative stress markers during ischemia, with improved protection at a lower concentration compared to the classical antioxidant MPG. Prolonged ischemic insult strongly reduced KH176m and MPG protection, probably because the amount of KH176m present within the ischemic heart was insufficient to prevent maximal oxidative stress levels to be reached during ischemia. Knowing that 4-HNE is mainly formed during ischemia [20, 21], our data indicates that antioxidant therapy in our model is mainly protective when it is able to attenuate the generation of oxidative stress markers during ischemia.

## Study Limitation

Our study illustrates the influence of ischemic duration on KH176m and MPG cardiac protection, but further research is needed to better understand the differences between mild and severe ischemia at a molecular level. Moreover, the equivalence between duration of ischemia in our isolated mouse heart model and that occurring in patients with acute myocardial infarction has not been clarified yet. Further studies are needed to make the direct translation of our findings to humans possible. Large animal studies will be necessary before finalizing the obtained results of the current work to humans. Considering that most patients with acute myocardial infarction receive reperfusion relatively late in real-world condition, the results of our studies showing effectiveness of antioxidant therapy at shorter but not longer ischemic duration in mice may possibly explain the frequently observed unresponsiveness of the so far used antioxidant interventions in humans. Short ischemia was effectively treated with KH176m. In the current study, we administered antioxidants before ischemia, which is relevant for clinical scenarios such as elective cardiac by-pass surgeries that are also known to cause ischemia-reperfusion injury to the heart. However, for treatment of acute myocardial infarct patients by PCI procedures, antioxidants administered during ischemia or early reperfusion should be tested to build up further understanding.

## Supplementary Information

ESM 1 (DOCX 1681 kb)

## Data Availability

The datasets used and/or analyzed in the current study are available from the corresponding author upon reasonable request.

## Declarations

All applicable international and/or institutional guidelines for the care and use of animals were followed. This article does not contain any studies with human participants performed by any of the authors.
