# Supplementary material for: The Redox Modulating Sonlicromanol Active Metabolite KH176m and the Antioxidant MPG Protect Against Short-Duration Cardiac Ischemia-Reperfusion Injury
Source: Cardiovasc Drugs Ther. 2021 Apr 29;35(4):745–58. doi: 10.1007/s10557-021-07189-9 (PMC8266721; doi:10.1007/s10557-021-07189-9)

**SUPPLEMENTARY FILES**

**The redox modulating sonlicromanol active metabolite KH176m,
 and the antioxidant MPG, protect against
short duration cardiac ischemia-reperfusion injury**

Yang Xiao^1*^, Karen Yim^1*^, Hong Zhang^1^, Diane Bakker^1^, Rianne Nederlof^2^, Jan A M Smeitink^3^, Herma Renkema^3^, Markus W Hollmann^1^, Nina C Weber^1^, Coert J Zuurbier^1^

^1^ Amsterdam UMC, University of Amsterdam, Laboratory of Experimental Intensive Care and Anesthesiology, Department of Anesthesiology, Amsterdam Cardiovascular Sciences, Meibergdreef 9, Amsterdam, The Netherlands

^2^ Institut für Herz- und Kreislaufphysiologie, Heinrich- Heine- Universität Düsseldorf, Universitätsstraße 1, Düsseldorf, Germany

^3^ Khondrion, Philips van Leydenlaan 15, Nijmegen, The Netherlands

*Contributed equally

Short Title: KH176m protects against short duration cardiac IR injury

Address for correspondence:

Coert J Zuurbier

Department of Anesthesiology, Amsterdam UMC

Meibergdreef 9, 1105 AZ

Amsterdam, The Netherlands

Phone: +31 (0) 205665259

Email: c.j.zuurbier@amsterdamumc.nl

Supplementary Information

**Supplementary Fig.1**


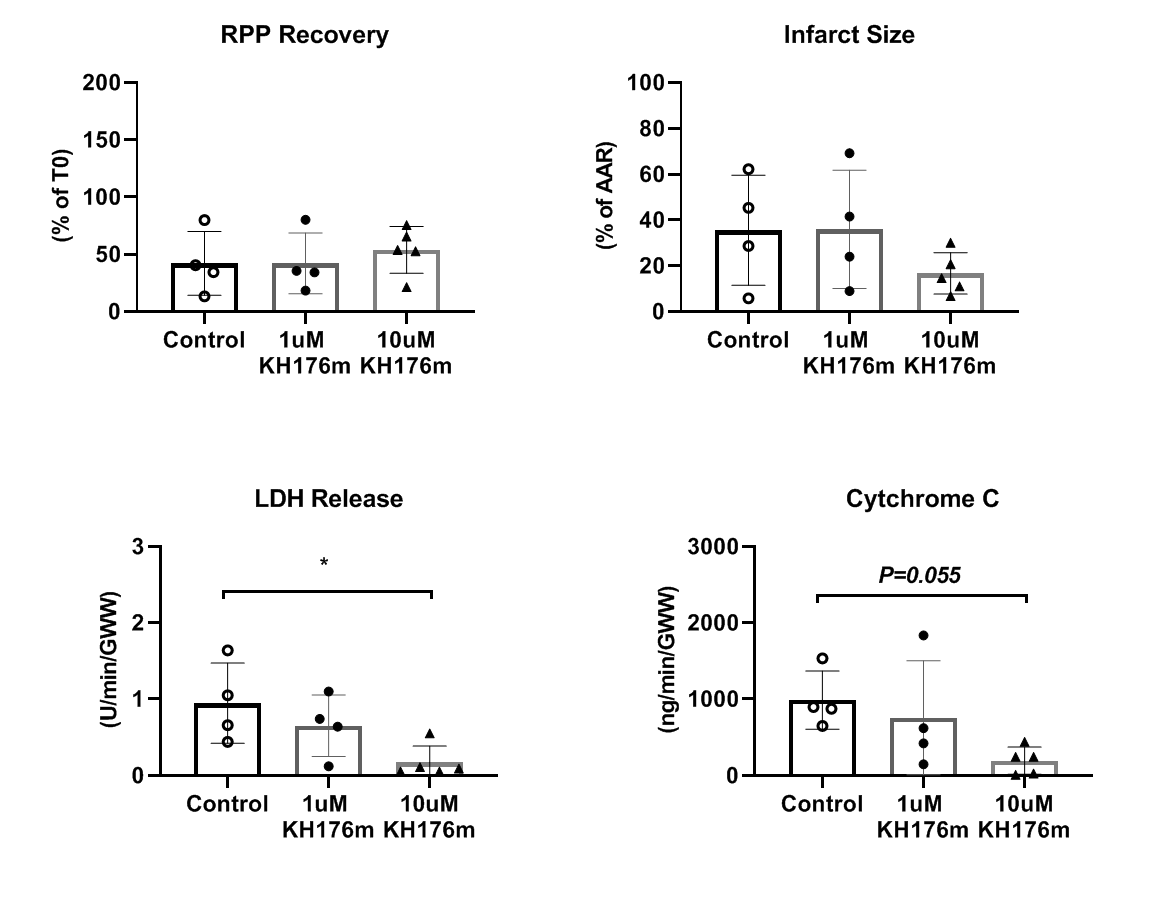


**Supplementary Fig. 1 Pilot experiment for different dosage of KH176m** The experiment followed mild IRI protocol described above. Heart was subjected to Saline (as control group), KH176m (1 µM) or KH176m (10 µM). (A) rate pressure product (RPP) recovery was calculated as the percentage of RPP at 60 min reperfusion to RPP at T= 0 min; (B) Infarct size related to area at risk (AAR); (C) Cytochrome c release at 10 min reperfusion, normalized to coronary flow; (D) LDH release at 10 min reperfusion, normalized to coronary flow. *P<0.05 vs Control group by one-way ANOVA with Dunnett’s post hoc vs. Control group.

**Supplementary Table 1**

Baseline characteristics at T0 for isolated mouse hearts subjected to both mild and severe IRI protocol

|  | 20min Ischemia | | | 30min Ischemia | | |
| --- | --- | --- | --- | --- | --- | --- |
|  | Control | 10µM KH176m | 1mM MPG | Control | 10µM KH176m | 1mM MPG |
| Pperf | 83±5 | 83±7 | 81±6 | 81±5 | 82±7 | 83±5 |
| EDP | 3.2±1.1 | 3.1±0.6 | 3.6±1.5 | 3.2±1.7 | 3.1±0.7 | 3.0±0.6 |
| DLVP | 131±19 | 132±13 | 134±24 | 141±22 | 143±8 | 143±13 |
| HR | 341±35 | 328±18 | 335±26 | 357±23 | 356±36 | 345±24 |
| RPP | 44939±8516 | 43491±5157 | 44844±7622 | 50171±6061 | 50796±4467 | 49247±5604 |
| +dp/dt | 5425±924 | 5561±731 | 5821±946 | 5909±1434 | 6007±437 | 5896±889 |
| -dp/dt | 4270±555 | 4489±485 | 4453±710 | 4405±713 | 4743±269 | 4713±499 |
| BW | 25.7±1.4 | 25.2±1.9 | 25.7±2.3 | 26.9±1.9 | 27.4±2.2 | 26.7±0.9 |
| Flow | 14.0±2.4 | 14.7±1.5 | 17.3±4.7 | 16.4±2.0 | 16.9±2.8 | 16.9±5.2 |
| T (°C) | 36.7±0.1 | 36.8±0.1 | 36.7±0.1 | 36.8±0.1 | 36.8±0.1 | 36.8±0.1 |

No differences were observed in baseline myocardial function within the intervention in the mild and severe ischemic injury. *Pperf*: perfusion pressure (mmHg); *EDP*: end diastolic pressure (mmHg); *DLVP*: developed left ventricular pressure (mmHg); *HR*: heart rate (bpm); *RPP*: rate pressure product (=DLVP * HR); *+dp/dt*: maximum contraction rate of left ventricle (mmHg/s); *-dp/dt*: maximum relaxation rate of left ventricle (mmHg/s); *BW*: body weight (gram); *Flow*: coronary flow (ml/min/GWW): *GWW*: heart wet weight (gram); *T*: temperature. Data are presented as Mean ± SD.

**Supplementary Table 2**

Specified area of risk and infarction

|  | 20min Ischemia | | | 30min Ischemia | | |  |
| --- | --- | --- | --- | --- | --- | --- | --- |
|  | Control | 10µM KH176m | 1mM MPG | Control | 10µM KH176m | 1mM MPG | |
| AAR | 230933±30199 | 235733±20507 | 249338±22904 | 258462±25093 | 257270±27674 | 258256±28550 | |
| AI | 70682±44536 | 35646±21569 | 62539±35331 | 145323±32126 | 128153±39001 | 110341±48958 | |
| IS% | 31±20 | 15±8* | 24±12 | 56±12 | 49±12 | 42±18 | |

No differences were observed in AAR within the intervention in the mild and severe ischemic injury. *AAR*: Area at risk (whole heart); *AI*: Area of infarction; *IS%:* infarct size related to AAR. Data are presented as Mean ± SD. *P<0.05 vs Control group.

**Supplementary Table 3**

Baseline characteristics at T0 for isolated mouse hearts subjected to molecular characterization protocol

|  | Sham | 20min Ischemia | | 30min Ischemia | |
| --- | --- | --- | --- | --- | --- |
|  |  | Control | 10µM KH176m | Control | 10µM KH176m |
| Pperf | 81± 4 | 82± 4 | 83± 4 | 83± 3 | 81± 3 |
| EDP | 3.5± 0.5 | 3.2± 0.2 | 3.4± 0.4 | 3.4± 0.2 | 3.3± 0.5 |
| DLVP | 140± 12 | 142± 10 | 146± 11 | 136± 20 | 133± 20 |
| HR | 314± 14 | 336± 25 | 346± 61 | 338± 34 | 353± 22 |
| RPP | 43948± 4392 | 47652± 4652 | 50087± 7552 | 45696± 6472 | 47086± 8905 |
| +dp/dt | 5845± 719 | 6224± 774 | 6351± 838 | 5804± 1043 | 5685± 679 |
| -dp/dt | 4516± 599 | 4756± 310 | 4905± 519 | 4615± 684 | 4549± 580 |
| BW | 27.2± 1.5 | 27.0± 1.5 | 26.7± 1.7 | 26.6± 2.2 | 28.5± 2.5 |
| Flow | 2.3± 0.6 | 2.2± 0.4 | 2.2± 0.2 | 2.4± 0.5 | 2.2± 0.3 |
| T (°C) | 36.9± 0.1 | 36.9± 0.1 | 37.0± 0.1 | 36.9± 0.1 | 36.9± 0.1 |

No differences were observed in baseline myocardial among these five groups. *Pperf*: perfusion pressure (mmHg); *EDP*: end diastolic pressure (mmHg); *DLVP*: developed left ventricular pressure (mmHg); *HR*: heart rate (bpm); *RPP*: rate pressure product (=DLVP * HR); *+dp/dt*: maximum contraction rate of left ventricle (mmHg/s); *-dp/dt*: maximum relaxation rate of left ventricle (mmHg/s); *BW*: body weight (gram); *Flow*: coronary flow (ml/min/GWW): *GWW*: heart wet weight (gram); *T*: temperature. Data are presented as Mean ± SD.

Image of coomassie blue staining:


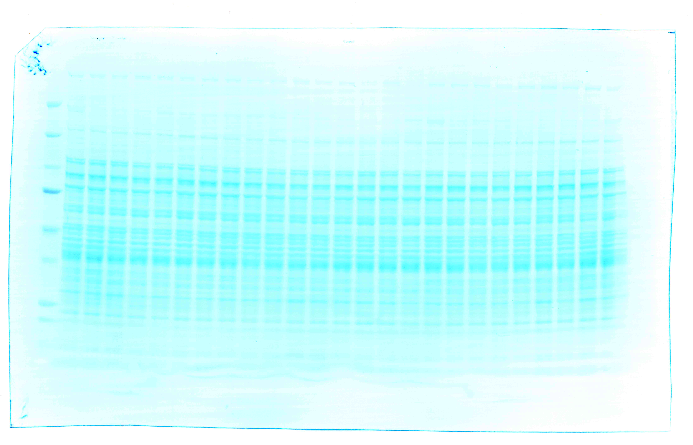

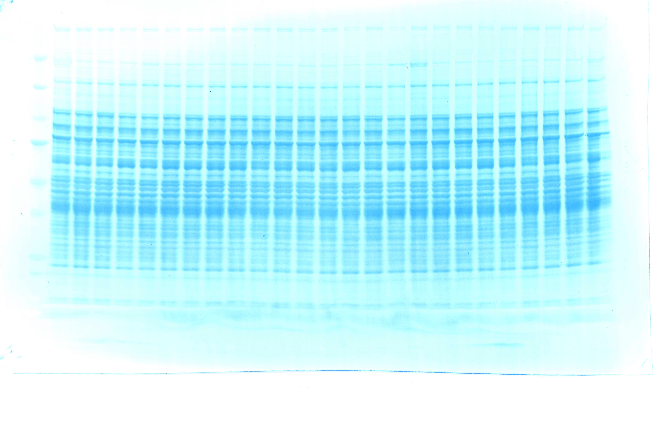


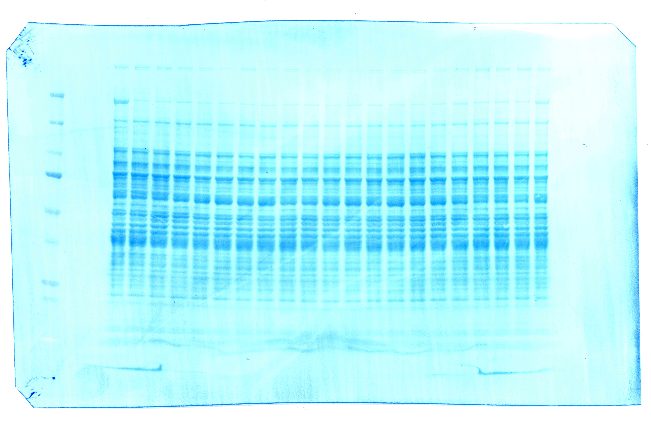

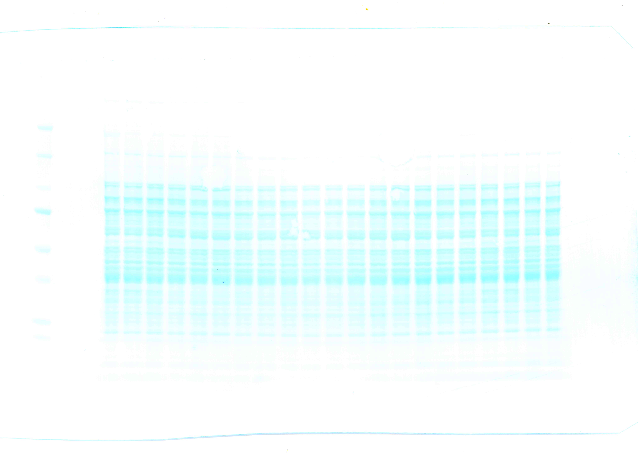


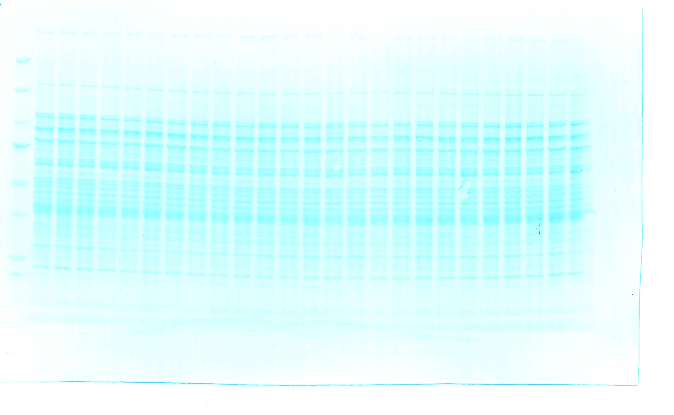

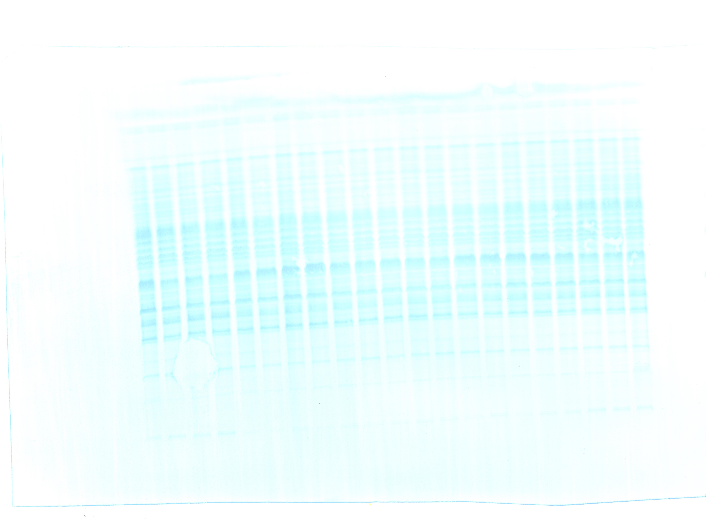

Supplement: Supplementary file 1 — (DOCX 1681 kb) [file 10557_2021_7189_MOESM1_ESM.docx]
